# Etymologia: *Mycobacterium chelonae*****

**DOI:** 10.3201/eid1709.ET1709

**Published:** 2011-09

**Authors:** Nancy Männikkö

**Affiliations:** Author affiliation: Centers for Disease Control and Prevention, Atlanta, GA, USA

**Keywords:** Etymologia, Mycobaterium chelonae

##  [mi′′ko-bak-tēr-eəm che′lō-nae]

From the Greek *mycēs, *fungus, *baktērion*, little rod, and *chelōnē*, turtle. German researcher Friedrich Freidmann reported isolation of this pathogen from the lung tissues of sea turtles (*Chelona corticata*) in 1903, referring to it as the turtle tubercle bacillus. In 1920, the Society of American Bacteriologists recommended that the organism be named after its discoverer, or *Mycobacterium friedmannii*. Bergey et al., however, chose in 1923 to instead recognize the host animal in the first edition of Bergey’s Manual of Determinative Bacteriology and listed the bacterium as *Mycobacterium chelonei*. The spelling was changed in the 1980s to *chelonae* to make it consistent with general use.

Sources: Dorland’s Illustrated Medical Dictionary. 31st ed. Philadelphia: Saunders; 2007; Grange JM. Mycobacterium chenolei. Tubercle. 1981;62:273–6.PubMed; Topley & Wilson’s Microbiology and Microbial Infections. Bacteriology, 10th ed., Vol. 2. London: Hodder Arnold; 2005.

